# Retraction: Panepoxydone Targets NF-kB and FOXM1 to Inhibit Proliferation, Induce Apoptosis and Reverse Epithelial to Mesenchymal Transition in Breast Cancer

**DOI:** 10.1371/journal.pone.0296553

**Published:** 2023-12-29

**Authors:** The PLOS ONE Editors

Following the publication of this article [1], concerns were raised regarding results in multiple figures. Specifically,

- In Figure 5, the following panels appear similar despite representing different conditions:
  - Figure 5A, MDA-MB-468 D-2 and D-3, and MDA-MB-231 D-3 panels.
  - Figure 5A, MDA-MB-453 D-1 and D-2 panel flipped horizontally.
  - Figure 5B, MDA-MB-468 Control and D-1 panels.
  - Figure 5B, MDA-MB-231 Control and MDA-MB-453 D-1 panels.
  - Figure 5B, MDA-MB-231 D-1 and D-2 panels.
- In Figure 3A, the vertical gate in MDA-MB-468 D-1, D-2, D-3 panels does not appear to be in the same location as the vertical gate in the corresponding Control panel.
- The β-actin panel for Bax and Bcl-2 in MDA-MB-453 cells in Figure 3C appears similar to the β-actin panel for MDA-MB-231 cells in Figure 4A despite representing different conditions.

PLOS attempted to discuss these issues with the authors but did not receive a response to our inquiries.

In light of the concerns affecting multiple figure panels that question the reliability and integrity of these data, the *PLOS ONE* Editors retract this article.

SM agreed with the retraction and apologized for the issues with the published article. RA, CY, BDG, MT, YX, ER, GAP, LBO, and WDC either did not respond directly or could not be reached.
